# Abortion Following the Administration of Chloroform

**Published:** 1856-02

**Authors:** 


*Abortion following the Administration of Chloroform.* By L. G. Robinson, M. D., of Detroit, Mich.—Some of the zealous advocates for the anæsthetic effects of chloroform, in the practice of midwifery, claim for that agent, efficiency of specific action upon the contractile fibre of the uterus. And in the divirsity of experience and observation, which is found among writers respecting its utility, it has obtained a "duplicity of character," or specific energy, which, if founded in truth, must have a *rationale* in some law of physiology. Whatever may be gathered from practical observation, contributing to the exposition of that governing law or principle, will be of interest to the profession, just in proportion to the amount of evidence fairly deduced from facts. Therefore, I offer the following case, the detail of which may have some weight worthy of record:

A few days since I was summoned to the bedside of Mrs. N——, an English lady, aged about 35, whose history is briefly as follows:—About four years since she came to this country, and soon after her arrival, became a resident of this city. She had been married six years, was the mother of two children, and had uniformly enjoyed excellent health. In appearance, she possesses a robust, vigorous constitution, of plethoric habit, and florid complexion—true to the English model. Nearly two months after her arrival in this city, she aborted in the 3rd month of pregnancy, the cause of which was attributed to a fall, and mechanical injury. She recovered promptly, however, and about two years since gave birth to a fine boy, at the end of a full period of gestation. Her labor was natural and easy, and her convalescence was not protracted. Since that time she has enjoyed uninterrupted good health up to the event of her present miscarriage.

On the morning of the day in which this occurred, she engaged in the duties of her household affairs, as well as usual, with the single exception of a slight toothache. Her sister, living in the adjoining house, had, for some time past, been in the habit of using chloroform by inhalation, quite freely, to relieve a facial neuralgia; and happening in the house of Mrs. N——, found her suffering, as she believed, from a similar cause, whereupon she immediately procured her bottle, and dropping a quantity upon her handkerchief, urged her to "snuff freely until she would begin to feel happy." Leaning back in her rocking chair, she gratified her sister by full inspirations until the toothache was forgotten, and even sensibility to *severe pinches*, so much obtunded as to afford great merriment for the sympathising and provident sister. She remained in this condition nearly a half hour, and it was even then most difficult to arouse her to a semi-conscious state, in which she expressed a desire to lie down on her bed. When asked if she was comfortable, and free from pain, she answered in the affirmative. In this happy dream she rested till about four o'clock P. M., when full consciousness returned, and on attempting to rise from her bed, vomited freely, and then was seized with pains of labor, more severe, tenfold, than she had ever suffered before. This had continued little more than an hour when I arrived. She at once informed me that she was in labor, (a fact clearly inferred from her expulsive efforts and intense suffering,) and, as she expressed it, "much before time," being in the fifth month of gestation.

Upon examination, I found uterine effort extremely severe, and continuous, while the fœtus was just passing from a well-relaxed os uteri, and was removed without delay, exhibiting no signs of viability. The after-pains continued severely intense, in spite of anodynes, for about six hours, accompanied with considerable hemorrhage and febrile excitement. But towards morning, of the following day, she awoke from a refreshing sleep of two hours' length, quite free from pain; and from that time has continued to convalesce without any untoward symptoms worthy of note.

Taking Dr. Snow (quoted by Ramsbotham) for authority, we have *five stages* or "*degrees*" in the pathological effects produced by Chloroform, as follows:

Firstly, there exists a kind of inebriation, which is usually agreeable when induced for curiosity. In the second degree, the mental functions are impaired, but not entirely suspended; consciousness, however, no longer continues correct, and a sort of dreamy state supervenes. "This degree may be considered analogous to delirium, and to certain states of the patient in hysteria and concussion of the brain; and it corresponds with that condition of an inebriated person, who is *not dead drunk*, but in the state described by the law as *drunk and not incapable*." It is very transitory, and if the inhalation be suspended, the patient, in a very few minutes, recovers the perfect possession of the mind. A considerable degree of anæsthesia is induced in this stage, and sometimes a high amount of mental excitement, that renders the patient difficult to manage, shows itself. In the third degree, all voluntary motion is paralysed. The fourth degree brings with it relaxation of the *voluntary* muscles, together with complete insensibility to external impressions, so that no pain is felt, even on the infliction of severe personal injuries. Yet, although reflex movements cannot be excited by touching even the most sensitive parts of the frame, still some functions of the spinal cord remain, as the sphincters continue contracted, and according to most of its advocates, *the action of the uterus in labor is not materially interfered with.* The fifth degree of narcotism is *the commencement of dying.*"

From these, Ramsbotham gives us the following synopsis of physiological phenomena exhibited in these effects:—

At *first* the *sensor* and *motor ganglia* are brought under its influence. The function of the cerebrum is next arrested, and *coma* supervenes, "a total abolition of consciousness, reducing life to a series of automatic movements." Then the *medulla oblongata* and the *spinal centres* becomes involved; and lastly, the *ganglionic system*, when the action of the heart is arrested, and life can no longer be supported.

This account of the various symptoms presented, and the order of their arrangement, may be correct as a "*general rule;*" but a margin must be left for the great exceptions growing out of the great diversity of constitutions, rendered peculiar by age, sex, temperament, diseased condition, etc., and, therefore, furnishing a corresponding variety in the degree of susceptibility to the influence of that agent. But let us apply our knowledge of its effects, so far as it goes, in the case before us. We find that the description (given by the sister of Mrs. N———,) of the effect, corresponds accurately with that of Dr. Snow's *second degree*, including, also, the abolition of sensibility described in his *fourth* degree. There can be no doubt that the patient was thoroughly under its influence, for she affirms that she has no recollection of getting from her chair to the bed, a distance of about ten feet. She can only recall the circumstances of inhaling the vapor.

The *rationale* proposed, with regard to the physiological action of chloroform, applied to this case, would be, that the first effect was *stimulant*, exciting the *sensor* and *motor ganglia*. Secondly, the function of the cerebrum was arrested, and partial *coma* supervened. Thirdly, the *medulla oblongata* and the *spinal centres* became involved, producing a *sedative* effect, relaxing the voluntary muscles, and, also, the os uteri. The stimulant effect first passed off, leaving the patient relaxed, or under the *sedative* influence. And her return to consciousness brought with it *re-action*, which ended when labor was completed. Now, *caution*, with regard to our conclusions, suggests the following inquiry, viz:—Would as profound inebriation from the effects of spirituous liquors, in *the same constitution*, have produced like effects? Would their sedative effect relax the voluntary muscles, and the os uteri, in a constitution that had been aborted? If so, then we can with reason suppose that re-action might be sufficient to introduce labor by exciting contractions of the uterus.

Where, then, in the history of this case, can Dr. Simpson, or any other advocate for the use of the anæsthetic effects of Chloroform in labor, point to its "*specific energy*" expended upon the contractile fibre of the uterus?

May we not as reasonably explain the phenomena produced without ascribing to it this peculiar property?

If we should jump at conclusions in our research for the *cause*, we should probably find many who would endorse the unqualified assumption that the *direct exciting cause* was chloroform. But such "circumstantial evidence," though apparently conclusive, ought not to be regarded as sufficient basis for even an assumed verity, respecting the physiological effect of that vapor.

Let us be content, therefore, for the present, with the simple record of the facts, and give them no more weight of evidence than that to which they are justly entitled, ever remembering that we are dealing with *the truths of Medical Science.—Peninsular Jour. Med.*

